# Comparative Evaluation of the Flexural Strength of Heat-Activated Polymethyl Methacrylate Denture Base Resin With and Without 0.2% by the Weight of Silver Nanoparticles Cured by Conventional and Autoclave Methods: An In Vitro Study

**DOI:** 10.7759/cureus.62675

**Published:** 2024-06-19

**Authors:** Kala Sukumaran, Smitha Ravindran

**Affiliations:** 1 Dentistry, Government Dental College, Thiruvananthapuram, IND; 2 Prosthodontics, Government Dental College, Thiruvananthapuram, IND

**Keywords:** polymethyl methacrylate, autoclave processing, denture base, water bath curing, silver nanoparticle, flexural strength

## Abstract

Purpose: Heat-activated polymethyl methacrylate (PMMA) is the most common and widely accepted denture base material. Two important drawbacks are the development of denture stomatitis and the high incidence of fracture of denture bases. The present study investigated the effect of adding 0.2% by weight of silver nanoparticles (AgNps) and using the autoclave method of terminal boiling on the flexural strength of heat-activated PMMA denture base resin.

Methods: A total of 40 samples of heat-activated PMMA blocks were divided into four groups, with 10 samples (n = 10) in each group. Group 1 consisted of unmodified heat-activated PMMA resin (PMMA-1) polymerized by the conventional method of terminal boiling (conventional curing); Group 2 consisted of 0.2% by weight AgNPs added to heat-activated PMMA resin (PMMA-2) polymerized by conventional curing; Group 3 consisted of PMMA-1 polymerized by the autoclave method of terminal boiling (autoclave curing); and Group 4 consisted of PMMA-2 polymerized by autoclave curing. The flexural strength was tested using a universal testing machine. Descriptive statistics were expressed as mean ± SD and median flexural strength. Kruskal-Wallis ANOVA with Mann-Whitney U post hoc test was applied to test for statistical significance between the groups. The level of significance was set at p<0.05.

Results: The results showed a statistically significant reduction in flexural strength in Group 2 compared to Group 1. The samples from Group 4 showed a statistically significant increase in flexural strength compared to Group 2. The Group 4 denture base had the highest flexural strength (115.72 ± 7.27 MPa) among the four groups, followed by Group 3 (104.16 ± 4.85 MPa). The Group 1 samples gave a flexural strength of 101.45 ± 3.13 MPa, and Group 2 gave the lowest flexural strength (85.98 ± 3.49 MPa) among the four groups tested.

Conclusion: The reduction in flexural strength of the heat-activated PMMA denture base after adding 0.2% by weight of AgNP as an antifungal agent was a major concern among manufacturers of commercially available denture base materials. It was proved in the present study that employing the autoclave curing method of terminal boiling for the polymerization of 0.2% by weight of AgNp-added heat-activated PMMA denture base resulted in a significantly higher flexural strength compared to the conventional curing method of terminal boiling for polymerization. Unmodified heat-activated PMMA gave higher flexural strength values when polymerized by autoclave curing compared to the conventional curing method of terminal boiling.

## Introduction

There has been an increase in the overall life expectancy of the elderly in recent decades due to developments in the healthcare system, leading to a high proportion of the aged population in the community. Most of them are partially or completely edentulous and require different types of prosthodontic treatment. Heat-activated polymethyl methacrylate (PMMA) has been the most commonly used denture base material worldwide for decades because of its low cost, simple processing technique, stable colors, optical properties, adequate strength, and other physical properties [1]. Denture stomatitis is a common, multifactorial infectious, inflammatory, and hyperplastic condition that is primarily caused by poor oral and denture hygiene, and full-time, mainly night-time, denture wear, bringing about the emergence of advanced *Candida*-containing polymicrobial biofilms near the host’s mucosal tissues [2]. Patients are advised to remove the denture at night and clean it manually and chemically to interrupt the biofilm formation. However, most complete denture wearers are old and have age-related disabilities that restrict proper cleaning of the surface of the dentures. This leads to the colonization of microorganisms that promote an inflammatory reaction in the oral mucosa. It would be beneficial if the denture base material could prevent biofilm formation or microbial adhesion [3]. New developments related to denture materials focus on reducing adhesive biofilm formation. These may have value in reducing bacterial and yeast colonization and could lead to reductions in denture stomatitis with appropriate denture hygiene.

This can be achieved by incorporating antimicrobial agents like silver nanoparticles (AgNPs) into the denture base [4]. Several studies have proved that adding AgNPs provided antifungal and antibacterial properties to denture base resins and denture lining materials, reduced biofilm formation, and effectively prevented denture stomatitis [5-8]. The large surface area of AgNP makes it highly reactive; it can diffuse through different biological membranes and provide a bacteriocidal effect due to the inhibition of cell multiplication and damage to bacterial cells [9]. A study by Vaiyshnavi et al. [10] proved that 0.2% by weight of AgNP incorporated heat-cured PMMA processed by injection molding technique provided maximum antifungal effect. Koroglu et al. [11] evaluated the mechanical properties of denture base resins after adding different concentrations of AgNP. They found that as the concentration of AgNPs increased, the flexural strength of the denture base decreased. The repetitive masticatory load on the denture base results in the stress concentration and propagation of cracks that weaken the denture base, resulting in fracture. So, the denture base should possess high flexural strength to resist the complex forces during mastication.

Indian researchers have extensively investigated the pressure cooker polymerization technique for processing heat-activated PMMA denture bases [12]. Durkan et al. conducted an in vitro study to compare conventional and autoclave curing techniques and found that autoclave polymerization resulted in superior transverse strength of the PMMA denture base resin [13]. In 2019, Gad [14] et al. investigated the comparative effects of different polymerization techniques and proved that the autoclave polymerization method of heat-cured PMMA was easy and resulted in superior flexural and surface properties. A comparison of the adaptation accuracy of maxillary complete dentures processed using different polymerization techniques showed that the autoclave processing technique increased the internal adaptation of the heat-cured PMMA denture bases [15]. Fouda et al. [16] proved that autoclave polymerization significantly increased the flexural strength, impact strength, and hardness of unmodified PMMA and 0.5% nanodiamond-added PMMA groups compared to the conventional polymerization technique.

Since it was proved in the earlier studies that 0.2% by weight AgNP incorporation into the denture base was effective against biofilm formation and preventing denture stomatitis [10], the alteration in flexural strength on the incorporation of 0.2% AgNP by weight and the effect of the autoclave method of terminal boiling on flexural strength were investigated in the present study. The primary objective was to find the difference in flexural strength of heat-activated PMMA (unmodified heat-activated PMMA) polymerized using the conventional method of terminal boiling and the autoclave method of terminal boiling. The secondary objective was to find the difference in the flexural strength between unmodified heat-activated PMMA and 0.2% AgNP-added heat-activated PMMA polymerized using the conventional and autoclave methods of terminal boiling. The present study aimed to determine and compare the flexural strength of unmodified heat-activated PMMA and 0.2% by weight of AgNPs added to heat-cured PMMA processed by conventional and autoclave curing methods of terminal boiling for polymerization of the denture base resin. 

## Materials and methods

A protocol to conduct an in vitro study on the comparative evaluation of the flexural strength of heat-activated PMMA with and without 0.2% by weight AgNP cured by conventional and autoclave methods was submitted before the institutional review committee of Government Dental College Thiruvananthapuram, India, and permission was obtained as per approval number DCT/IEC/SS/24/23. The details of the materials used in the study are given in Table 1.

**Table 1:** List of materials used in the present study APS: average particle size; CAS: Chemical Abstract Service Registry (US); SSA: specific surface area

Sl No	Material used	Batch details/Specification	Manufacturer
1.	Alginate impression material: Algitex	2245; date of manufacture: February 2024	Dental Products of India, Mumbai, Maharashtra, India
2.	Modeling wax: Hindustan modelling wax no. 2	2324; date of manufacture: January 2023	The Hindustan Dental Products, Hyderabad, Telangana, India
3.	Plaster of Paris: Ramaraju	POPO 37/24; date of manufacture: February 2024	The Ramaraju Surgical Cotton Mills Limited, Virudhunagar, Tamil Nadu, India
4.	Cold mold seal: isolate	JR/M/MB/020785; date of manufacture: February 2022	Prevest DenPro Limited, Jammu, Jammu and Kashmir, India
5.	Polymethyl methacrylate (polymer): acryton -H- pink	HP3KGGP22011; date of manufacture: September 21, 2022	New India Dental Products, Bulandshahar, Uttar Pradesh, India
6.	Polymethyl methacrylate (monomer): acryton -H	HL4L22012; date of manufacture: September 21, 2022	New India Dental Products, Bulandshahar, Uttar Pradesh, India
7.	Nanosilver particles	Stock no.: VI/16/119; purity: 99.9%; APS: 10-100nm; CAS: 7440-22-4; SSA: 15-22m^2^/g	Vedayuky India Private Limited, Jamshedpur, Jharkhand, India

**Figure 1:**
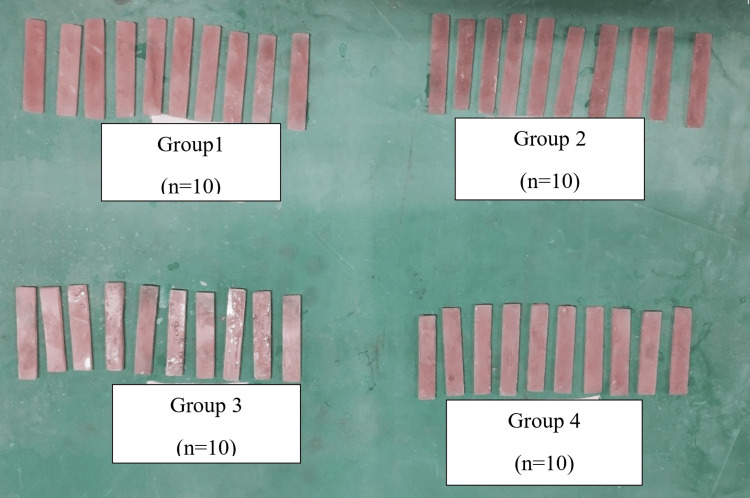
Heat-activated PMMA samples prepared in four groups for the flexural strength testing in the present study Group 1: PMMA-1 (unmodified heat-activated PMMA) polymerized by the conventional method of terminal boiling (conventional curing); Group 2: PMMA-2 (0.2% by weight of AgNP-added heat-activated PMMA) polymerized by the conventional curing method; Group 3: PMMA-1 polymerized by the autoclave method of terminal boiling (autoclave curing); Group 4: PMMA-2 polymerized by the autoclave curing method PMMA: polymethyl methacrylate; AgNP: silver nanoparticle

The sample size was estimated using N Master software 2.0 (Informer Technologies Inc., Los Angeles, CA) based on the values of a previous study [16] in which the flexural strength measurement of unmodified heat-activated PMMA following the conventional method of terminal boiling and the autoclave method of terminal boiling was done. On substituting the values, the sample size obtained was four (n = 4). In the present study, the sample size was taken as 10 (n = 10) per group. The compression molding technique [17] was used for preparing four groups of heat-activated PMMA specimens, as shown in Figure 1. A metal pattern was made with a dimension of 64 x 10 x 3.3 mm according to International Organization for Standardization (ISO) standards 2013 [18]. The alginate impression of the metal pattern was made, and the wax pattern was prepared by pouring the molten wax into the alginate impression. The wax pattern was invested in a denture flask using model plaster, following the manufacturer's instructions for water-powder ratio, mixing time, and setting time. Dewaxing was carried out after the final set of the model plaster. A cold mold seal (isolate) was brushed on the surface of the mold. The test samples with heat-cured PMMA were prepared using the mold space obtained. The samples of Group 1 and Group 3 were prepared using unmodified heat-activated PMMA (PMMA-1). A powder-liquid ratio of 100 grams of unmodified heat-activated PMMA (powder) and 40 grams of monomer (liquid) was used [19] for preparing PMMA-1. The samples of Group 2 and Group 4 were made by heat-activated PMMA with 0.2% AgNP by weight (PMMA-2), which was prepared by adding 0.28 grams of AgNP to a mixture of 100 grams of unmodified heat-activated PMMA (powder) and 40 grams of monomer (liquid). The amount of AgNP needed was calculated as 0.2/100 x (100 + 40) = 0.28 grams of AgNP.

**Figure 2:**
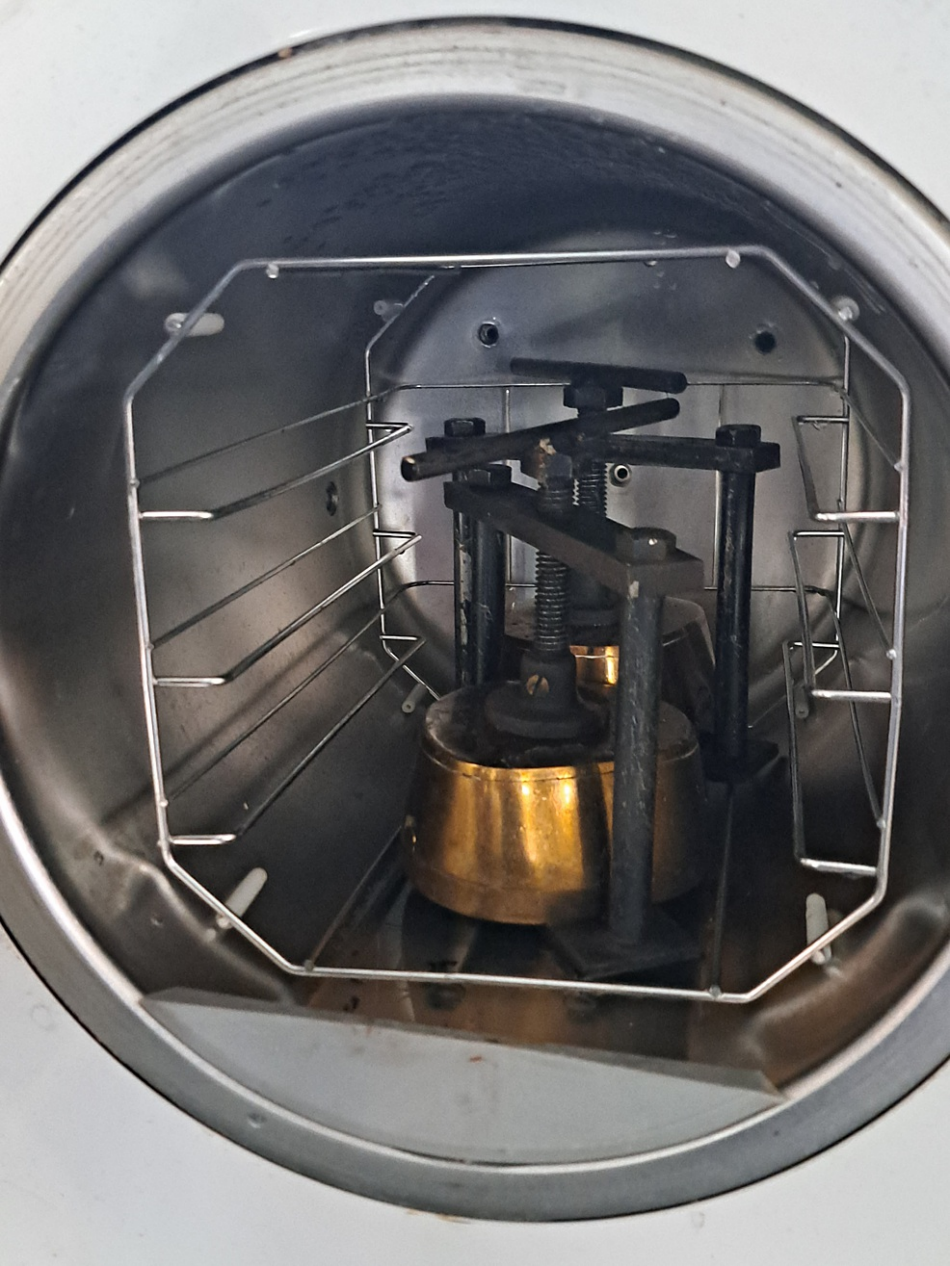
Denture flasks kept inside the pre-heated autoclave for autoclave curing

**Figure 3:**
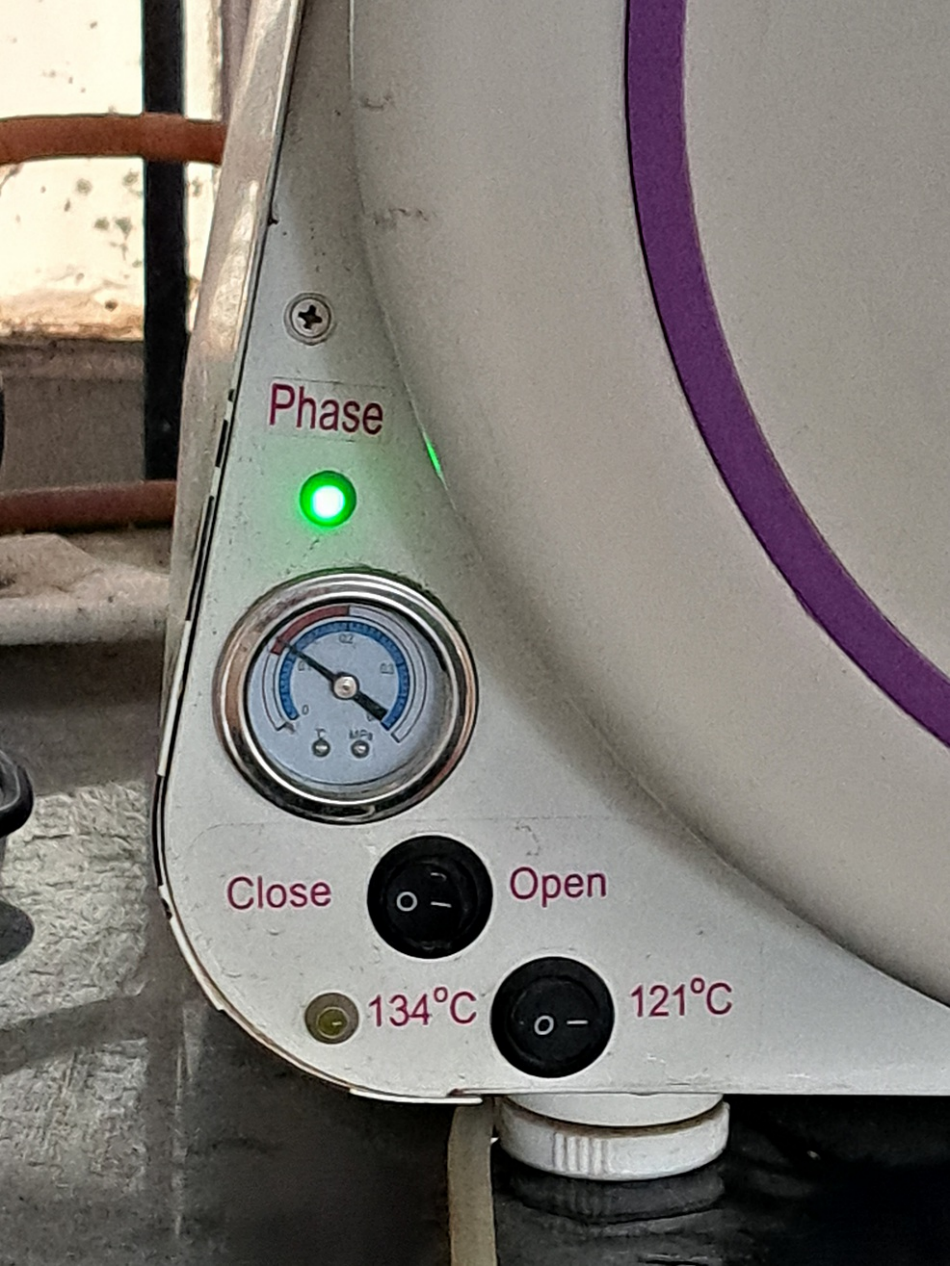
The preprogrammed cycle of the autoclave at 121°C and 0.12 MPa pressure for autoclave curing

The material was mixed following the manufacturer's instructions and packed at the dough stage. Trial closure was done, and denture flasks were clamped. The flasks were kept for one hour for bench curing, and the polymerization procedure was carried out. The denture flasks with Group 1 samples were kept in a thermostatically controlled water bath at room temperature. The temperature was raised to 74°C and kept for two hours, and terminal boiling was done at 100°C for one hour (conventional curing) [17]. Group 2 samples packed in the denture flasks in the dough stage were processed by the same conventional curing method as that of Group 1. The samples of Group 3 were bench-cured for one hour after packing, and the flasks were kept in a thermostatically controlled water bath at room temperature. The temperature was raised to 74°C and kept for two hours. After that, the flasks were transferred to a preheated autoclave (Figure 2) and subjected to terminal boiling at 121°C at 0.12 MPa pressure for 25 minutes, as shown in Figure 3 (autoclave curing), similar to the study conducted by Fouda et al. [16]. A fully automatic autoclave DOLPHINN Steam Sterilizer (Woson Medical Instrument Co., Ltd, Ningbo, China) was used in the present study. Group 4 samples were packed in the dough stage, bench-cured, and processed similarly to Group 3. After the programmed cycle, the pressure was released, the door was opened, and the clamped flasks were removed. All the flasks were opened after 24 hours. The excess was trimmed with acrylic trimming stones, and polishing was done with sandpaper with various grits, followed by pumice. A caliper was used to verify the length, width, and thickness of each sample, and the samples were stored in distilled water for 48 hours.

**Figure 4:**
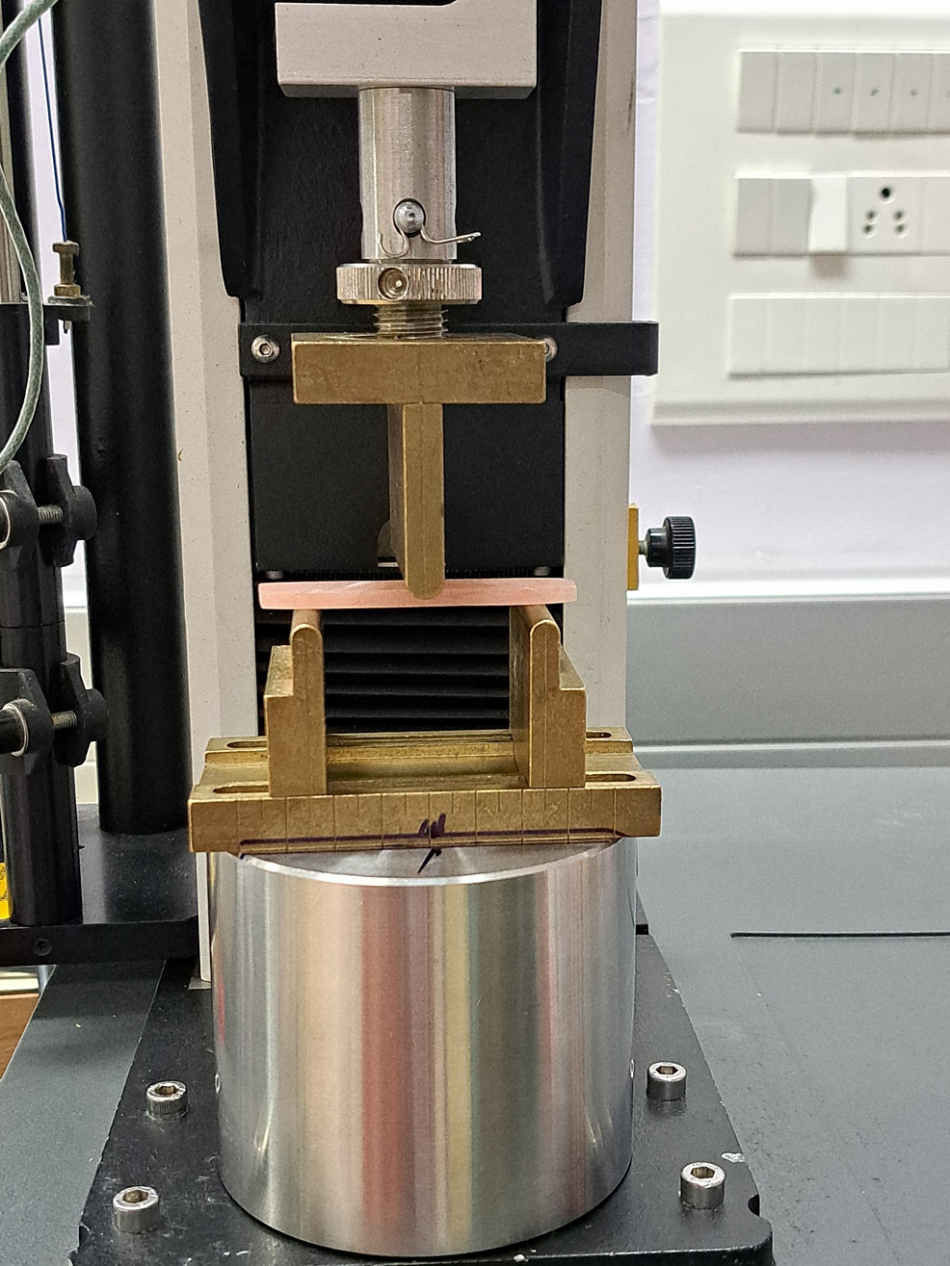
The specimen was placed on two supports 50 mm apart, and a three-point bending test was done by applying a load from the top.

The samples of the four groups were tested for flexural strength by subjecting each sample to a three-point bending test using a Universal Testing Machine (Instron, Norwood, MA). Each specimen was positioned on two parallel supports with a 50 mm span width and a 50 kg load applied at 5 mm/minute crosshead speed [16]. The bending force was applied to the middle of the specimen, as shown in Figure 4. The load at which the fracture occurred was recorded. The flexural strength (σ) was calculated in Newton/square millimeters (N/mm^2^) or MPa using the formula [16]: σ = 3PL / 2wt^2^ where P = load at fracture, L = distance between two supports (span width in mm), w = width of the specimen in mm, and t = thickness of the specimen in mm.

The observed data were coded, tabulated, and analyzed using IBM SPSS Statistics software for Windows, version 20 (IBM Corp., Armonk, NY). Descriptive statistics were expressed as mean ± SD and median flexural strength. Due to a smaller sample size of 10, non-parametric tests (Kruskal-Wallis ANOVA followed by a Mann-Whitney U post hoc test) were applied to test statistical significance between groups. The level of significance was set at p<0.05.

## Results

The difference in flexural strength between PMMA-1 and PMMA-2 polymerized by the conventional method of terminal boiling (conventional curing) and autoclave method of terminal boiling (autoclave curing) was investigated. Table 2 shows the mean, standard deviation, and median of the flexural strength of the study groups. The samples of autoclave-cured PMMA-2 gave the highest mean flexural strength, and conventional-cured PMMA-2 gave the lowest mean flexural strength. The result of the Kuskal-Wallis ANOVA test is given in Table 3. A statistically significant difference (p<0.05) in flexural strength was observed between the groups studied. The results of the pairwise analysis of the groups are given in Table 4. There was a statistically significant difference (p<0.05) in flexural strength between PMMA-1 and PMMA-2 on polymerization using conventional curing, between PMMA-1 conventional curing and PMMA-2 autoclave curing, between PMMA-2 conventional curing and PMMA-1 autoclave curing, between PMMA-2 conventional curing and PMMA-2 autoclave curing, and between PMMA-1 and PMMA-2 while employing autoclave curing. The PMMA-1 gave higher flexural strength values following autoclave curing than conventional curing but was not statistically significant.

**Table 2:** Mean, standard deviation, and median flexural strength of Group 1, Group 2, Group 3, and Group 4 samples PMMA-1: unmodified heat-activated PMMA resin (heat-activated PMMA resin without AgNP); PMMA-2: heat-activated PMMA resin with 0.2% by weight AgNP; conventional curing: conventional method of terminal boiling for polymerization; autoclave curing: autoclave method of terminal boiling for polymerization PMMA: polymethyl methacrylate

Group	Mean ± SD	Median
Group 1 (PMMA 1: conventional curing)	101.45 ± 3.13	99.99
Group 2 (PMMA 2: conventional curing)	85.98 ± 3.49	86.07
Group 3 (PMMA 1: autoclave curing)	104.16 ± 4.85	104.23
Group 4 (PMMA 2: autoclave curing)	115.72 ± 7.27	116.79

**Table 3:** Comparison of flexural strength of Group 1, Group 2, Group 3, and Group 4 heat-activated PMMA resin PMMA-1: unmodified heat-activated PMMA resin (heat-activated PMMA resin without AgNP); PMMA-2: heat-activated PMMA resin with 0.2% by weight AgNP; conventional curing: conventional method of terminal boiling for polymerization; autoclave curing: autoclave method of terminal boiling for polymerization A p-value less than 0.05 (p<0.05) is considered significant. PMMA: polymethyl methacrylate

Group	Median	Mean Rank	p-value
Group 1 (PMMA 1: conventional curing)	99.99	19.30	< 0.001*
Group 2 (PMMA 2: conventional curing)	86.07	5.50	
Group 3 (PMMA 1: autoclave curing)	104.23	23.10	
Group 4 (PMMA 2: autoclave curing)	116.79	34.10	

**Table 4:** Post-hoc (pairwise) analysis (Mann-Whitney U test) of the flexural strength of PMMA test specimens PMMA-1: unmodified heat-activated PMMA resin (heat-activated PMMA resin without AgNP); PMMA-2: heat-activated PMMA resin with 0.2% by weight AgNP; conventional curing: conventional method of terminal boiling for polymerization; autoclave curing: autoclave method of terminal boiling for polymerization A p-value less than 0.05 (p<0.05) is considered significant. PMMA: polymethyl methacrylate

Group	N	Mean rank	Median	p-value
Group 1 (PMMA 1: conventional curing)	10	15.50	99.99	< 0.001*
Group 2 (PMMA 2: conventional curing)	10	5.50	86.07	
Group 1 (PMMA 1: conventional curing)	10	8.90	99.99	0.226
Group 3 (PMMA 1: autoclave curing)	10	12.10	104.23	
Group 1 (PMMA 1: conventional curing)	10	5.90	99.99	0.001*
Group 4 (PMMA 2: autoclave curing)	10	15.10	116.79	
Group 2 (PMMA 2: conventional curing)	10	5.50	86.07	< 0.001*
Group 3 (PMMA 1: autoclave curing)	10	15.50	104.23	
Group 2 (PMMA 2: conventional curing)	10	5.50	86.07	< 0.001*
Group 4 (PMMA 2: autoclave curing)	10	15.50	116.79	
Group 3 (PMMA 1: autoclave curing)	10	6.50	104.23	0.002*
Group 4 (PMMA 2: autoclave curing)	10	14.50	116.79	

## Discussion

Most denture wearers are geriatric patients with age-related restrictions that interfere with the proper maintenance of acrylic dentures, leading to denture stomatitis. It was proven earlier that 0.2% by weight of AgNP-added heat-activated PMMA denture base prevented or delayed the development of denture stomatitis. The effect of adding 0.2% by weight of AgNP on flexural strength was investigated in the first part of the study. It was found that the flexural strength of heat-activated PMMA denture base resin decreased significantly when 0.2% by weight AgNP was added and polymerized by the conventional curing method. Similar results were obtained earlier in the study conducted by Alla et al. [3].

Heat-activated PMMA has been used as the permanent denture base material for several decades. According to ISO 20795-1:2013, heat-activated PMMA is classified as Type 1, Class 1 polymerizable denture base material, which contains powder and liquid [18]. When the powder and liquid are mixed, they pass through three steps, namely, initiation, propagation, and termination, resulting in the formation of polymer chains [20]. The conversion of methyl methacrylate to PMMA denture base is a free radical addition polymerization reaction [21], and the heat-activated benzyl peroxide is the initiator that provides free radicals. A cross-linking agent like ethylene glycol dimethacrylate (EGDMA) connects the linear polymer chains, resulting in a cross-linked polymer with superior mechanical properties [17]. On heating, the linear polymer chains are formed at around 70°C, and cross-linking happens at around 100°C during the terminal boiling [22]. 

Flexural (transverse) strength is a combination of compressive, tensile, and shear strengths, and it represents the stiffness and resistance of a material to fracture [23]. The higher the degree of polymerization and cross-linking, the superior the mechanical properties of the denture base. The polymerization rate is directly proportional to the initiator efficiency, and during the polymerization of AgNP-added heat-activated PMMA, the AgNP acts as a scavenger for the initiator, interfering with polymerization and adsorbing the active polymer chains, resulting in a decreased diffusion rate [24]. This results in a decrease in the number of polymer chains produced and a considerable reduction in flexural strength. Since the reduction in flexural strength results in the fracture of the complete denture, AgNP cannot be added as an antifungal agent to the heat-activated PMMA if the processing is done using the conventional curing method for polymerization.

In the second part of the study, PMMA-1 in Group 3 and PMMA-2 in Group 4 were polymerized by the autoclave curing method of terminal boiling. The results showed that the flexural strength increased following the autoclave curing method compared to the conventionally cured groups. On comparing the flexural strength of PMMA-1 autoclave-cured samples with PMMA-1 conventional-cured samples, an increase in flexural strength was observed in the former group. On comparing the flexural strength of PMMA-2 autoclave-cured with PMMA-2 conventional-cured, a statistically significant increase in flexural strength was observed in the former group. So it is proven that 0.2% by weight AgNPs added to heat-activated PMMA denture base resin when processed by the autoclave method of terminal boiling results in a denture base with higher flexural strength compared to the denture base polymerized by the conventional method of terminal boiling.

The autoclave polymerization depends on the autoclave machine used in sterilization, which works by applying steam at high pressure and temperature. When the packed and clamped flask is transferred from a water bath at 74°C to an autoclave at 121°C, the rapid temperature rise produces more free radicals and ultimately more growing polymer chains. The free radicals collide with other radicals or polymer chains, increasing branching and cross-linking of the interstitial polymer [21]. The boiling point of the monomer is elevated due to the high pressure in the autoclave, and almost all the monomers will get polymerized before the monomer boils. As a result, the porosity due to the evaporation of the monomer is reduced. The high pressure accelerates the initial polymerization and increases the dispersal of the unreacted methyl methacrylate molecules into the PMMA matrix, resulting in increased polymerization and a decreased residual monomer level, thereby improving the flexural strength [25].

The two most common causes of denture base fractures are impact failure outside the mouth and flexure fatigue failure inside the mouth [26]. The ability to withstand multidirectional and intricate masticatory loads is the fundamental and essential requirement for a denture base material [27]. According to ISO 20795-1:2013, the minimal flexural strength required for denture bases is 65 MPa [18]. The residual ridge resorption is a continuous and irreversible process, and the denture will become loose over time. On mastication, the loose-fitting denture base will rock on the supporting ridges, and the cyclic masticatory loads will result in micro-cracks leading to fracture of the denture base over time. A denture base with more than the minimum required flexural strength is needed to provide more resistance to the forces of mastication and prevent fracture of the denture base. Therefore, continuous research is going on to find out the methods for improving flexural strength by altering the composition and processing method of heat-activated PMMA denture base resins.

Based on the findings of the present study, it can be concluded that the autoclave curing method of terminal boiling can be used for curing 0.2% by weight of AgNP-added heat-activated PMMA denture base resins without reducing the flexural strength. This method helps to achieve superior flexural strength properties of 0.2% by weight of AgNP-added heat-activated PMMA denture base resins and unmodified heat-activated PMMA denture base resins.

Limitations of the study

The impact strength, cytotoxicity, and biocompatibility of 0.2% by weight of AgNP-added heat-activated PMMA denture base resins were not included in the present study. These factors need to be evaluated before conducting clinical trials of a heat-cured PMMA denture base with 0.2% by weight of AgNP processed by the autoclave curing method of terminal boiling.

## Conclusions

The present study investigated the effect of adding 0.2% by weight of AgNP on the flexural strength of heat-activated PMMA denture base resin following the conventional method of terminal boiling and the autoclave method of terminal boiling. The flexural strength decreased when 0.2% by weight of AgNP was added and polymerized by the conventional method of terminal boiling. When heat-activated PMMA without 0.2% by weight of AgNP (unmodified heat-activated PMMA) was polymerized by the autoclave method of terminal boiling, the flexural strength increased but was not statistically significant. There was a statistically significant increase in the flexural strength of 0.2% by weight AgNP-added heat-activated PMMA when polymerized by the autoclave method of terminal boiling compared to the conventional curing method.
